# Cultivation of *Bartonella henselae* in an *Ixodes ricinus* cell line to assess this tick as a potential reservoir of the bacterium

**DOI:** 10.2478/jvetres-2025-0045

**Published:** 2025-09-01

**Authors:** Violetta Zając, Jacek Sroka, Ewa Bilska-Zając, Angelina Wójcik-Fatla

**Affiliations:** 1 Department of Health Biohazards and Parasitology, Institute of Rural Health, Lublin, 20-090 Poland; 2 Department of Parasitology and Invasive Diseases, Bee Diseases and Aquatic Animal Diseases, National Veterinary Research Institute, Puławy, 24-100 Poland

**Keywords:** arthropod cell culture, Bartonellaceae, cat-scratch disease, tick-borne disease, vector competence

## Abstract

**Introduction:**

*Bartonella* spp. are gram-negative, facultative intracellular bacteria with zoonotic potential. These microorganisms are emerging vector-borne pathogens distributed worldwide and infecting humans, domestic mammals and wildlife. This study investigated the possibility of culturing *Bartonella henselae* in a tick cell line derived from *Ixodes ricinus*.

**Material and Methods:**

The *Ixodes ricinus* embryonic cell line (IRE/CTVM19) and the Houston-1 strain of *B. henselae* were used for culture studies. Replication of *B. henselae* was quantified with the use of a SYBR Green real-time PCR and transcribed complementary DNA (cDNA) in samples collected separately from the supernatant and monolayer of culture from 1 to 9 days post-infection (d.p.i.). Identification of *B. henselae* was based on the detection of a fragment of the *ribC* gene encoding riboflavin synthase. Quantification was performed indirectly by determining the threshold cycle.

**Results:**

Microscopic observations confirmed that infection with *B. henselae* did not show any visible negative effect on tick cells. The quantity of *B. henselae* cDNA from the monolayer remained low, and a slight increase was observed at 4, 8 and 9 d.p.i. Significantly, the highest amount of *B. henselae* was observed at 2 d.p.i. in samples isolated from the supernatant.

**Conclusion:**

The maintenance of live *B. henselae* in an *I. ricinus*-derived cell line was confirmed. The low level of multiplication in the tick cell line suggested a limited role of *I. ricinus* as a reservoir of *B. henselae*. The IRE/CTVM19 tick cell line is suitable for culture of *B. henselae*, and this model may be useful in further studies.

## Introduction

The *Bartonella* genus belongs to the Bartonellaceae family, Rhizobiales order and Alphaproteobacteria class and comprises Gram-negative, facultative intracellular bacteria with zoonotic potential. *Bartonella* species are emerging vector-borne pathogens spreading worldwide and infecting humans, domestic mammals and wildlife (11, 13, 18). At least 17 out of 40 described *Bartonella*species and subspecies are associated with various clinical manifestations in humans and animals (11), from a mild flu-like illness to more severe ones such as myocarditis, endocarditis, arthritis and hepatitis (13). The severity of clinical symptoms depends mainly on the patient’s immune status, but other factors such as the species of infecting pathogen, virulence factors and bacterial load may also have an impact (8).

*Bartonella henselae* is the aetiological agent of cat-scratch disease (CSD), the most common bartonellosis in humans, manifesting with lymphadenopathy and fever after being scratched or bitten by a cat. Less frequently reported are hepatic lesions, ocular disease, osteomyelitis and endocarditis. Symptoms of *Bartonella quintana* infection, causing trench fever mainly among the homeless population, may include acute febrile or subacute endocarditis (13, 21).

*Bartonella* species show high adaptation to one or several reservoir hosts: *B. henselae* is adapted to cats, *B. vinsonii* subsp. *berkhoffii* to canids and *B. bovis* to cattle (13). Among domestic animals, cats are the main reservoirs of *B. henselae*, as well as of *B. koehlerae* and *B. clarridgeiae*. One of the risk factors associated with *B. henselae* infection in cats was lack of tick control (20). Infection of dogs, which are likely accidental hosts, was confirmed for *B. henselae, B. vinsonii* subsp. *berkhoffii, B. koehlerae, B. clarridgeiae, B. elizabethae, B. washoensis, B. quintana, B. bovis, B. volans*-like, and *B. rochalimae*. Two species pathogenic to humans, *B. melophagi* and *B. alsatica*, were identified in sheep and rabbits, respectively (8). Among wildlife, *B. rochalimae* was detected in foxes and wolves, Candidatus *B. merieuxii* and *B. vinsonii* subsp. *berkhoffii* also in wolves (11) and *B. schoenbuchensis* and *B. bovis*in roe deer and red deer (1). The greatest diversity of *Bartonella* species occurs among small mammals, and the most common species they carry are *B. grahamii, B. elizabethae, B. tribocorum, B. taylorii* and *B. queenslandensis* (18).

*Bartonella bovis, B. schoenbuchensis* and *B. chomelii* were found in bovine reservoirs (8). In a study conducted in Nigeria, a DNA fragment of a *gltA* gene confirmed as *B. bovis* was detected in 9.3% of cattle blood samples, mainly from animals slaughtered for human consumption. The authors suggested more research to validate their results and determine how poor sanitation and insufficient meat inspection in Nigeria exacerbate the impact on public health of the prevalence of *B. bovis* (15). In a study conducted in Vietnam on food rats which had been trapped in different ecosystems, *Bartonella* spp. was detected in 14.9% of blood samples tested. Three of the five identified species (*B. elizabethae, B. rattimassiliensis* and *B. tribocorum*) had zoonotic potential (19).

*Bartonella* spp. are transmitted by various arthropod vectors, mainly sandflies, lice, fleas, keds, mites and possibly ticks (8, 13). These bacteria are most often transmitted through the faeces of fleas and lice and bites of mosquitoes, and potentially may be through the bites of infected ticks (24). Recent reports suggested that ticks may serve as potential vectors of *Bartonella* spp. The DNA of *Bartonella* spp. was detected in different tick species collected all over the world, including that of *B. henselae* in *Ixodes ricinus* (22, 27). Cases of mainly CSD patients infected with *Bartonella* after tick bites and without contact with cats, and high seroprevalence of *Bartonella* spp. in a population occupationally exposed to tick bites were also described (2, 8).

The aim of this study was to investigate the possibility of culturing *B. henselae* in a tick cell line derived from *I. ricinus*. We hypothesised that the growth of bacteria in tick cells *in vitro* could prove the role of *I. ricinus* as a reservoir of *B. henselae*.

## Material and Methods

### Tick cell line

The *Ixodes ricinus* embryonic cell line (IRE/CTVM19) (5, 16), derived from ticks collected in the UK, was provided by the Tick Cell Biobank (University of Liverpool, Liverpool, UK). The tick cell line was maintained according to the protocol provided by the Tick Cell Biobank and described by Zając *et al*.(28). The cells were propagated in L-15 (Leibovitz) medium supplemented with 20% foetal bovine serum, 10% tryptose phosphate broth, 2 mM L-glutamine, 100 U/mL penicillin and 100 μg/mL streptomycin at pH = 7.4 (all from Sigma Aldrich, St. Louis, MO, USA) in flat-sided culture tubes (Nunc, Thermo Fisher Scientific, Waltham, MA, USA) and in ambient air at 28°C. The medium was changed once a week by removal and replacement of three-quarters of the volume. Subcultures were performed monthly by adding an equal volume of fresh medium to the tube, resuspending the cells by pipetting and transferring half of the suspended cells and medium into a new tube. Before subculturing, the cells were checked under an inverted microscope to confirm their expected morphology and sufficiently high density.

### Infection of the tick cell line with *B. henselae*

**Fig. 1.**
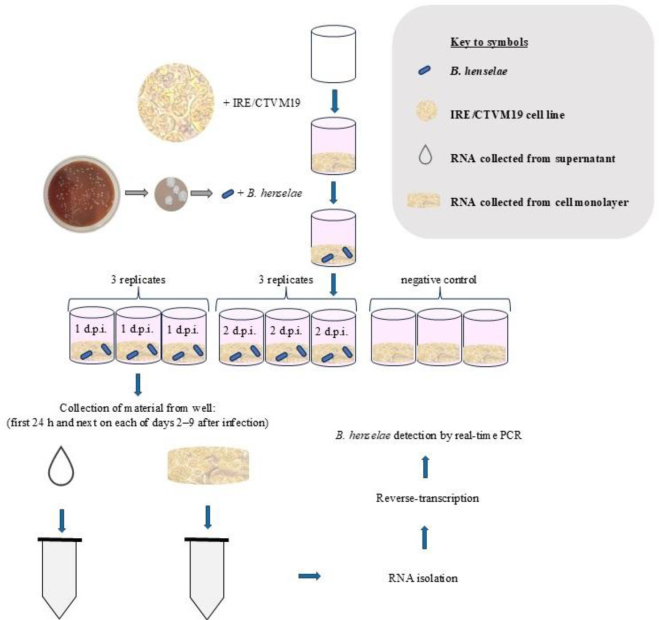
Experimental design schematic. d.p.i. – days post infection; B. *henselae* – *Bartonella henselae*; IRE/CTVM19 – *Ixodes ricinus* embryonic cell line

The Houston-1 strain of *Bartonella henselae*(ATCC 49882) isolated from an HIV-positive male was purchased from the American Type Culture Collection (ATCC, Manassas, VA, USA). *B. henselae* was cultured onto tryptic soy agar (TSA – Sigma Aldrich) supplemented with 5% defibrinated sheep blood (Biomaxima, Lublin, Poland) and incubated on plates at 37°C in a humidified atmosphere of 5% CO_2_. Subcultures were started every two weeks by the transferring of single colonies onto fresh plates. For inoculation of the tick cell line, ten-day post-subculture bacteria (passage 8) were used. Before the experiment, the IRE/CTVM19 cells were seeded at a density of 7.0–7.4 × 10^5^ cells per mL in 1 mL of total complete medium without antibiotics in 24-well plates (Nunc, Thermo Fisher Scientific) and maintained at 28°C overnight for attachment of cells. On the next day, 27 wells were infected with 100 μL of *B. henselae*suspension in tryptic soy broth (TSB – Sigma Aldrich) with 5% defibrinated sheep blood (Biomaxima) at a density of four colonies of *B. henselae* growing on TSA suspended in 1 mL of TSB, and were incubated in ambient air at 28°C. The entire contents of three infected wells each day post infection (d.p.i), were harvested separately as the supernatant and the monolayer for RNA extraction. This took place daily on days 1–9 after infection with *B. henselae*. After collection of supernatant, the bottom of the well was scraped into 600 μL of RLT Buffer (Qiagen, Hilden, Germany) using an RNAse-free sterile tip, pipetted and collected into a sterile tube (Fig. 1). The uninfected culture (negative control) consisted of three additional wells seeded with IRE/CTVM19 cells as described above that were not infected with bacteria. From those wells, the material was not harvested for RNA extraction but was used to compare uninfected tick cells with those infected with bacteria for all days post infection in daily observation under an inverted microscope.

### Isolation of RNA, reverse transcription and real-time PCR

Ribonucleic acid was extracted immediately after the collection of material from the well using an RNeasy Mini Kit (Qiagen), according to the manufacturer’s protocol for animal cells. The isolated RNA was stored at −80°C (ULTF 80; Arctiko, Esbjerg N, Denmark). In the next step, the total RNA was reverse-transcribed into complementary DNA (cDNA) with a QuantiTect Reverse Transcription Kit (Qiagen). The concentrations of RNA and cDNA were measured in individual samples using a spectrophotometer (NanoDrop; Thermo Fisher Scientific). The concentration of isolated RNA in the tested samples ranged from 2.5 to 4.9 ng/μL for samples of supernatant and from 123 to 230 ng/μL for the cell monolayer, and the cDNA concentration ranged from 990 to 1,250 ng/μL. Quantification of *B. henselae* in infected tick cell cultures was carried out by real-time PCR in samples of transcribed cDNA with a Power SYBR Green PCR Master Mix (Applied Biosystems, Paisley, UK). For the detection of *B. henselae,*a single primer pair as described by Johnson *et al*. (14) targeting the riboflavin synthase gene (ribC) was used: BAR1 (5ʹ-TAACCGATATTGGTTGTGTTGAAG-3ʹ) and BAR2 (5ʹ-TAAAGCTAGAAAGTCTGGCAACATAACG-3ʹ). Reactions were performed according to Hobson *et al*. (12) (with some modification) in a final volume of 10 μL, and the reaction mixture contained 5 μL of reaction buffer with SYBR Green, 2 μL of cDNA, 1.5 μL of nuclease-free water and 0.75 μL of each primer (10 mM). The reaction was carried out in a StepOne thermal cycler (Life Technologies Holding, Singapore) using the following cycle profiles: 95°C for 10 min; and 40 cycles of 95°C for 1 min, 60°C for 1 min and 72°C for 1 min. Each time, a positive control was used, which consisted of genomic DNA isolated from a culture of *B. henselae*on TSA, as was a negative control in which ultrapure water was used instead of cDNA. Quantification was performed indirectly by determining the threshold cycle (C_t_).

### Statistical analysis

The statistical significance of the differences in Ct values for individual samples across days post infection was evaluated using a two-sample *t*-test, and the significance of the differences across sample variants was determined using one-way analysis of variance. A P-value < 0.05 was considered statistically significant.

## Results

Microscopic observations of live IRE/CTVM19 cultures infected with *B. henselae* showed no visible negative effect of the bacterial infection on the tick cells. In the infected cultures, no tick cell lysis was observed, and the level of adhesion and morphology of tick cells remained unchanged for 9 d.p.i. compared to uninfected cultures assessed on the same day.

**Fig. 2.**
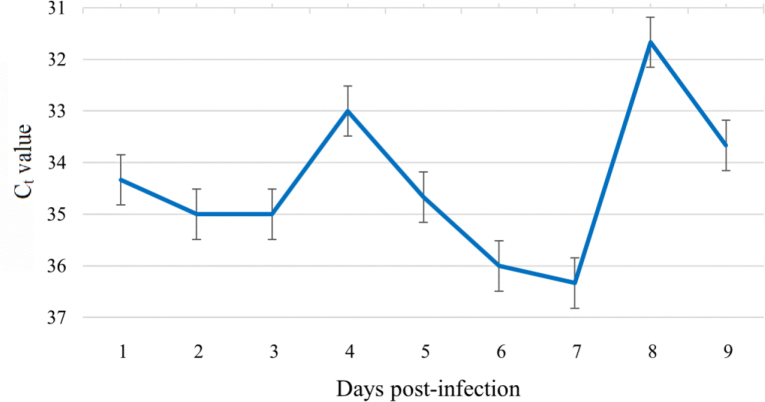
Quantification of *Bartonella henselae* complementary DNA assessed by threshold cycle (C_t_) values determined by real-time PCR in samples of monolayer cells from *Ixodes ricinus* embryonic cell line (IRE/CTVM19) cultures. Data were reported as the mean C_t_ ± standard error of the mean for three replicates of each day post infection

In samples collected from monolayer cells, the amount of *B. henselae* cDNA remained low (mean C_t_ value from 31.7 to 36.3) at 1–9 d.p.i., and on days 4 (C_t_ = 33), 8 (C_t_ = 31.7) and 9 (C_t_ = 33.7) only a slight increase was observed (Fig. 2). On days 6 and 7, the amount of cDNA was the lowest: C_t_ = 36 and 36.3, respectively (Fig. 2). The analysis did not show statistically significant differences in the C_t_ value between days post infection.

**Fig. 3.**
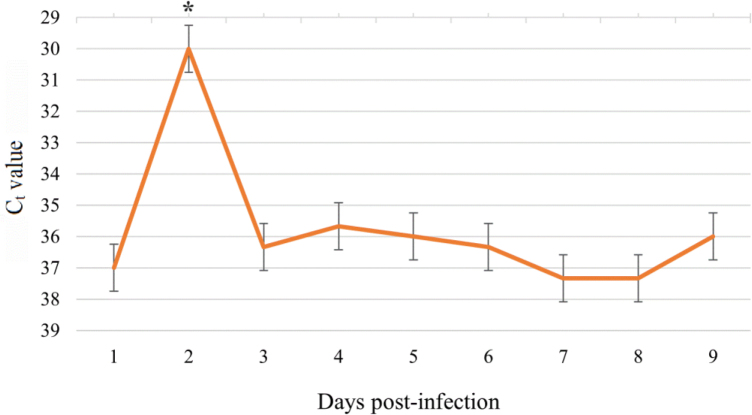
Quantification of *Bartonella henselae* complementary DNA assessed by threshold cycle (C_t_) values determined by real-time PCR in samples of supernatant cells from *Ixodes ricinus* embryonic cell line (IRE/CTVM19) cultures. Data were reported as the mean C_t_ ± standard error of the mean for three replicates of each day post infection. * – statistically significant (P-value < 0.01)

The bacterial cDNA quantity in samples collected from the culture supernatant at 1 d.p.i. was low and only sufficient for the Ct to be 37. In the monolayer samples at 2 d.p.i., the amount of *B. henselae* was the highest (C_t_ = 30; P-value < 0.01). In these samples from 3 to 9 d.p.i., it remained low: the average C_t_ value ranged from 35.7 to 37.3 (Fig. 3).

No statistically significant differences were found between Ct values assessed in monolayer and supernatant samples (P-value = 0.145). In each real-time PCR reaction, the C_t_ value result for the positive control (DNA isolated from *B. henselae* culture) was 23–24, and no amplification was observed for the negative control.

## Discussion

Tick cell lines have been widely used in the study of viral and bacterial infection, including studies of pathogen–tick interactions, gene expression and the biology and physiology of ticks, and for genetic manipulation (4, 16). Billeter *et al*. (6) used several *Bartonella* species to infect three tick cell lines derived from *Amblyomma americanum, Ixodes scapularis* and *Rhipicephalus sanguineus*. The authors observed a significant increase in the growth of two *B. henselae*isolates on days 2 or 3 post wash (at 4 or 5 d.p.i.). In our experiment, a significant increase of *B. henselae* cDNA in culture supernatant samples was demonstrated at 2 d.p.i., while in cell monolayer samples insignificant growth of bacterial cDNA was observed at 4 and 8 d.p.i. Our results from the monolayer samples are similar to those 4-d.p.i. and 2-day-post-wash results reported by Billeter *et al*. (6). Although Billeter *et al*. (6) used the whole contents of the well for DNA isolation, during the post-infection wash the bacteria from the supernatant were probably removed. In contrast, the increase obtained on day 8 could not be compared with any result of that study, which ended on day 5 post wash (7 d.p.i.). Both studies confirmed that *B. henselae* can replicate in tick cell lines. However, their results were difficult to compare day for day because of the differences in experimental designs. Billeter *et al*. (6) presented the results as isolate growth for each day post wash, which was a two-day time shift from each day post infection in our result presentation. The use of different tick-derived cell lines and *B. henselae* isolates may also have partly caused the discrepancy in results. Billeter *et al*. (6) also found different results in duplicate studies for two *B. henselae* isolates in the same laboratory conditions. In our study, we did not observe high replication of *B. henselae* in the tick cell line, except at 2 d.p.i. in supernatant samples. Since our studies were based on RNA, which is less stable than DNA, the loss of some RNA material during laboratory procedures also cannot be ruled out.

Billeter *et al*. (6) observed a high cytopathic effect of *B. henselae* infection on tick cells, which was not demonstrated in our experiment. In our study, we did not detect greater vacuolisation or lysis of infected cells compared to uninfected cells. The reason for such differences may be the use of a different tick cell line in the cited experiment to the line used in ours and the competence of vector species. Additionally, the inconsistent observation of cytopathic effect across the previous research and ours may be because genetic variations occur between different tick cell lines and even within the same line at different passages, as recent studies confirmed (16). This is due to the relatively unstable karyotype, which may influence the biology and physiology of tick cells. To confirm the results of both experiments, further studies are needed under the same conditions, taking the same amount of *B. henselae*used for inoculation and the same tick cell line and performing multiple repetitions.

Our study showed that the cDNA level of *B. henselae* in the supernatant samples increased only at 2 d.p.i. and remained low for the rest of the days. The supernatant of tick cell cultures received the inoculate in this experiment and was a medium where the bacteria could replicate, and from 3 d.p.i. to the end of the study, *B. henselae* were probably mostly within tick cells or attached to them. A previous study using electron microscopy visualised *B. henselae* as intracellular bacteria, clustered in morula-like vacuoles or distributed throughout the cytoplasm of tick cells and between the cells (6). However, we did not observe a correlation between the quantity of bacteria in the supernatant and the quantity in the monolayer; the high increase at 2 d.p.i. in the supernatant was not matched by a high decrease in the monolayer. Additionally, in samples of the monolayer we observed an increase of *B. henselae*cDNA at 4 d.p.i., followed by a decrease at 5–7 d.p.i. and an increase again at 8 d.p.i. The fluctuations of bacterial growth were probably related to the biology of invasion, entry to tick cells and release to the medium, but the exact mechanisms of *B. henselae* behaviour are unknown and need further investigation.

Transmission of *Bartonella* spp. by ticks is speculated but not confirmed (10). Cotté *et al*. (9) used *I. ricinus* ticks feeding on *B. henselae*-infected blood on artificial membranes to investigate transstadial and transovarial transmission, and by feeding the ticks subsequently on uninfected blood, to investigate further transmission to blood. Their study showed the competence of *I. ricinus* to transmit *B. henselae*, of which the viability was confirmed by culture. Transstadial transmission was also confirmed, as *B. henselae* was detected in nymphs and adult ticks after moulting from larvae and nymphs, respectively. These ticks fed on infected blood at their preceding stages. However, transovarial transmission was not confirmed. Bacterial DNA was detected in eggs laid by females, which fed on infected blood, but it was not detected in hatched larvae (9). A more recent study partially confirmed the above results, although *B. henselae* was also detected in larvae (17). Another study, examining *R. sanguineus* as a vector of *B. henselae*, suggested transstadial transmission and the ability of this tick species to retransmit bacteria during blood meals (25, 26). A study on *R. sanguineus* infected with *B. vinsonii* subsp. *berkhoffii* by capillary tube feeding confirmed the presence of bacterial DNA in ticks and their faeces; however, culture of viable bacteria was unsuccessful (7). The competence of *I. ricinus* to transmit *B. birtlesii* was confirmed in a study carried out on a murine model. In this study, the acquisition and maintenance of bacteria by the ticks and injection of the bacteria with tick saliva were demonstrated (23). In contrast, a study in the United States on a large group of *Ixodes scapularis* and *Ixodes pacificus* ticks concluded that these species were probably not involved in the transmission of *Bartonella* spp. (3).

Further investigations are needed to confirm whether the tick cells or some other biological components associated with them are necessary for bacterial multiplication. Although *B. henselae* can passively enter tick cells *via* endocytosis, it is still uncertain whether these bacteria can actively infect tick cells and if the presence of tick cells is required for their replication. As elements of research on the molecular mechanisms responsible for interactions between *B. henselae* and tick cells, *in vivo* studies including the immunological response of tick cells to infection with *Bartonella* species would be valuable.

## Conclusion

The presented results confirmed maintenance of live *B. henselae* in an *I. ricinus*-derived cell line. Low multiplication in the tick cell line suggested a limited role of *I. ricinus* as a reservoir of *B. henselae*.The IRE/CTVM19 tick cell line was suitable for *B. henselae* culture, and this model may be useful in further studies of the interaction between the pathogen and the tick.
